# Persistence of SARS-CoV-2 antibodies over 18 months following infection: UK Biobank COVID-19 Serology Study

**DOI:** 10.1136/jech-2023-220569

**Published:** 2023-11-03

**Authors:** Jelena Bešević, Ben Lacey, Howard Callen, Wemimo Omiyale, Megan Conroy, Qi Feng, Derrick W Crook, Nicola Doherty, Daniel Ebner, David W Eyre, Dan Fry, Edward Horn, E Yvonne Jones, Brian D Marsden, Tim E A Peto, Fenella Starkey, David Stuart, Samantha Welsh, Natasha Wood, Alan Young, Allen Young, Mark Effingham, Rory Collins, Jo Holliday, Naomi Allen

**Affiliations:** 1 Nuffield Department of Population Health (NDPH), University of Oxford, Oxford, UK; 2 Nuffield Department of Medicine (NDM), University of Oxford, Oxford, UK; 3 UK Biobank, Stockport, UK; 4 University of Oxford Big Data Institute, Oxford, UK

**Keywords:** INFECTIONS, COVID-19, PUBLIC HEALTH, EPIDEMIOLOGY

## Abstract

**Background:**

Little is known about the persistence of antibodies after the first year following SARS-CoV-2 infection. We aimed to determine the proportion of individuals that maintain detectable levels of SARS-CoV-2 antibodies over an 18-month period following infection.

**Methods:**

Population-based prospective study of 20 000 UK Biobank participants and their adult relatives recruited in May 2020. The proportion of SARS-CoV-2 cases testing positive for immunoglobulin G (IgG) antibodies against the spike protein (IgG-S), and the nucleocapsid protein (IgG-N), was calculated at varying intervals following infection.

**Results:**

Overall, 20 195 participants were recruited. Their median age was 56 years (IQR 39–68), 56% were female and 88% were of white ethnicity. The proportion of SARS-CoV-2 cases with IgG-S antibodies following infection remained high (92%, 95% CI 90%–93%) at 6 months after infection. Levels of IgG-N antibodies following infection gradually decreased from 92% (95% CI 88%–95%) at 3 months to 72% (95% CI 70%–75%) at 18 months. There was no strong evidence of heterogeneity in antibody persistence by age, sex, ethnicity or socioeconomic deprivation.

**Conclusion:**

This study adds to the limited evidence on the long-term persistence of antibodies following SARS-CoV-2 infection, with likely implications for waning immunity following infection and the use of IgG-N in population surveys.

**WHAT IS ALREADY KNOWN ON THIS TOPIC**

- The dynamics of SARS-CoV-2 antibodies following infection has been extensively assessed over the course of a year following infection.

**WHAT THIS STUDY ADDS**

- To the best of our knowledge, this study is the first to report the persistence of antibodies over an 18-month period following SARS-CoV-2 infection.

**HOW THIS STUDY MIGHT AFFECT RESEARCH, PRACTICE OR POLICY**

- Our findings indicate that over a quarter of individuals are unlikely to have detectable IgG-N antibodies at 18 months after infection. This has implications for immunity following infection, and the use of IgG-N antibodies in population surveys to avoid underestimating prevalence of past infection.

## Introduction

Since the emergence of SARS-CoV-2 in December 2019, there have been over 650 million confirmed cases of COVID-19 worldwide.^1^ Although vaccination is currently effective at reducing the risk of severe COVID-19, there is ongoing transmission of SARS-CoV-2, driven in part by waning immunity after infection or vaccination.

Following infection, most individuals will develop circulating antibodies to SARS-CoV-2.^2^ However, the long-term persistence of these antibodies has not been well described. Understanding the persistence of SARS-CoV-2 antibodies would improve our understanding of the protection afforded following infection and inform estimates of the likely trajectory of SARS-CoV-2 transmission, especially in populations with limited availability or uptake of vaccines.

We aimed to determine the duration of antibody response following SARS-CoV-2 infection over an 18-month period, and to assess whether this is affected by demographic factors such as age, sex and ethnicity.

## Methods

### Design and participants

UK Biobank participants, resident in the mainland UK in May 2020, were invited by email to join the UK Biobank COVID-19 Serology Study.^3^ Participants were asked whether they would be willing to take serial blood samples in their own home and answer brief questionnaires about potential COVID-19 symptoms. In order to increase the age range of the study, UK Biobank participants (who are now typically 50–80 years) were asked to forward an email invitation from UK Biobank to their adult (ie, ≥18 years old) children and/or grandchildren inviting them to join the study. UK Biobank participants and their adult relatives were then selected by random sampling, stratified by demographic characteristics, with some subsequent oversampling from ethnic minority groups and from urban settings. Efforts were made to select only one participant from a given address.

### SARS-CoV-2 antibody assessment

In the first phase of the study, participants were posted a self-sampling blood kit and symptom questionnaire every month for 6 months between the end of May and beginning of December 2020. Instructions on how to collect and return the sample (together with a link to an online information video) were included in all sampling kits. Participants were asked to return the sample (containing ~0.5 mL capillary blood) and questionnaire by post to the UK Biobank laboratory on the day of sample collection. Samples were tested for immunoglobulin G (IgG) antibodies against the spike protein (IgG-S) of the SARS-CoV-2 virus, determined using an ELISA platform at the Target Discovery Institute laboratory, University of Oxford.^4^ In the second phase of the study, participants were sent a final blood sample kit between November 2021 and February 2022. While the first phase of the study measured IgG-S antibodies, the second phase of the study involved testing blood samples for antibodies against the nucleocapsid protein (IgG-N) of the virus. The change in antibody test used to determine past infection was necessary as the UK vaccination programme began in December 2020 and after this date IgG-S antibodies, which can be present as a result of either infection or vaccination, could not be used to determine infection, whereas IgG-N antibodies, which are generated as a result of infection only, could be used to determine infection. Thriva test kits were used to collect the blood samples, which were subsequently analysed at a nominated Thriva laboratory.

### Statistical analysis

Participants with previous SARS-CoV-2 infection were identified by a positive antibody test or positive PCR test (available for existing UK Biobank participants via linkage with the national testing data). The date of infection was estimated as date of PCR or 21 days prior to first positive antibody test (accepting that in some participants the date of infection was likely earlier, but accounting for the lag in time between infection and the generation of detectable levels of antibodies). Individuals with evidence of reinfection, defined as a positive PCR test >90 days after the date of first SARS-CoV-2 infection,^5^ were excluded. To determine the seroprevalence of IgG-S antibodies following infection in the first phase of the study, the proportion of SARS-CoV-2 cases testing positive for IgG-S antibodies was calculated along with 95% CIs at the following time intervals: 30–60, 60–90, 90–120, 120–150 and 150–180 days following the date of infection. The denominators were restricted to cases that returned an analysable sample during the given time interval. The seroprevalence of IgG-N antibodies following infection was similarly calculated for the following time intervals: 30–270, 270–450 and 450–630 days. Heterogeneity was assessed using a χ^2^ test. Statistical analysis was performed in R V.4.1.1.

## Results

Overall, 20 195 individuals were recruited into the UK Biobank COVID-19 Serology Study. The median age of participants was 56 (IQR 39–68) years, 56% were female and 88% were of white ethnicity; 94% returned one or more blood samples (table 1; online supplemental table S1).

10.1136/jech-2023-220569.supp1 Supplementary data

**Table 1:** Characteristics of study participants

	All participants recruited		Participants in IgG-S analyses		Participants in IgG-N analyses	
	n	%	n	%	n	%
Age, years						
<30	2291	11	228	15	126	7
30–39	3169	16	243	16	168	10
40–49	2514	12	181	12	143	8
50–59	3928	20	338	22	505	29
60–69	4071	20	279	18	460	26
70+	4222	21	245	16	347	20
Gender						
Men	8877	44	636	42	764	44
Women	11 318	56	878	58	985	56
Ethnicity*						
White	17 626	88	1241	82	1391	80
Other ethnicity	2526	13	271	18	354	20
Townsend Deprivation Index†						
Less deprived	6971	35	453	30	571	33
Average	8249	41	590	39	715	41
More deprived	4975	25	471	31	463	27
UK region						
East Midlands	1201	6	76	5	90	5
East of England	938	5	53	4	60	3
London	6059	30	637	42	663	38
North East	832	4	44	3	63	4
North West	2175	11	167	11	205	12
Scotland	1227	6	60	4	70	4
South East	2604	13	166	11	193	11
South West	1379	7	65	4	87	5
Wales	791	4	37	2	56	3
West Midlands	1407	7	113	8	123	7
Yorkshire and the Humber	1582	8	96	6	139	8
All	20 195	100	1514	100	1749	100

IgG-S antibody analyses restricted to SARS-CoV-2 cases with one or more IgG-S tests following diagnosis. IgG-N antibody analyses restricted to SARS-CoV-2 cases with IgG-N test following diagnosis.

*Excludes participants of unknown ethnicity.

†Area-level derived measure of socioeconomic deprivation (categories are defined as: <−2 (less deprived), −2 to <2 (average), 2+ (more deprived)).

**Figure 1:**
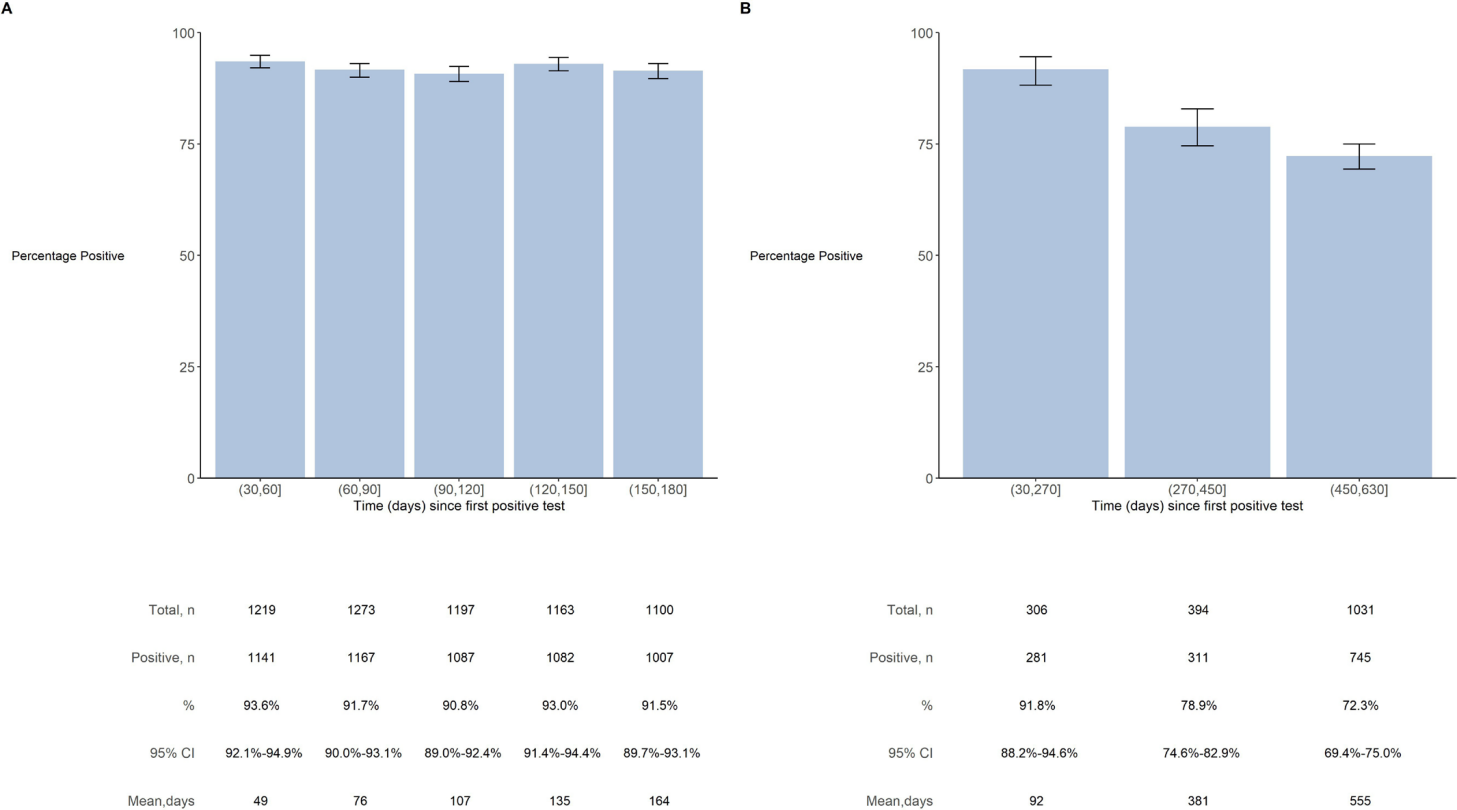
Proportion of SARS-CoV-2 cases seropositive for IgG-S and IgG-N antibodies over an 18-month period following infection. (A) Analyses among 1514 SARS-CoV-2 cases who had one or more subsequent IgG-S antibody tests; 74 individuals are omitted from the plot as they have a duration of follow-up less than 30 days and a further 27 individuals are omitted from the plot as they exceed 180 days duration. Proportions are % (95% CI) of IgG-S seropositive cases among participants that returned a valid test during each period; participants may contribute to more than period. (B) Analyses among 1749 SARS-CoV-2 cases with IgG-N test following diagnosis; 78 individuals are omitted from the plot as they have a duration of follow-up less than 30 days and a further 16 individuals are omitted from the plot as they exceed 630 days duration. Proportions are % (95% CI) of IgG-N seropositive cases among participants that returned a valid test during each period; participants provided one IgG-N test only.

In the first phase of the study, there were 1514 cases of SARS-CoV-2 infection with at least one subsequent IgG-S antibody test. The proportion of SARS-CoV-2 cases with IgG-S antibodies following infection remained high over the first 6 months, with ~92% (95% CI 90%–93%) of those tested between 150 and 180 days after infection having detectable antibodies (figure 1A). There was no strong evidence of heterogeneity in this proportion by age, sex, ethnicity or socioeconomic deprivation (online supplemental table S2).

There were 1749 cases of SARS-CoV-2 infection with a subsequent IgG-N antibody test in the second phase of the study. There was a progressive reduction in the proportion of individuals with detectable levels of IgG-N antibodies over 18 months following infection (figure 1B): 92% (95% CI 88%–95%) had IgG-N antibodies at about 3 months, 79% (95% CI 75%–83%) at 12 months and 72% (95% CI 70%–75%) at 18 months.

There was no strong evidence of heterogeneity in the persistence of Ig-N antibodies by age, sex, ethnicity or socioeconomic deprivation index; neither was there evidence that seroprevalence varied by method of case ascertainment, as similar estimates were observed at 18 months when limiting the analysis to cases identified via PCR test only (online supplemental table S3 and figure S1).

## Discussion

In this population-based cohort study, the proportion of SARS-CoV-2 cases with IgG-S antibodies following infection remained high at 92% at 6 months. Longer follow-up for IgG-N antibodies found a progressive reduction in the proportion of cases with detectable levels of antibodies over 18 months following infection, falling from 90% at 3 months to 79% at 12 months, and 72% at 18 months. There was no evidence of variation in findings for IgG-S or IgG-N by age, sex, ethnicity or socioeconomic deprivation.

Several previous studies on antibody dynamics following SARS-CoV-2 infection report broadly consistent findings to the present study at 1 year or less of follow-up, but there were no identified studies with 18 months of follow-up.^6–14^ One of the largest studies, of nearly 40 000 individuals from the USA, found that IgG-S and IgG-N antibodies were detected in ~90% and ~70% of individuals, respectively, at 10 months after infection; however, there was substantial heterogeneity in the IgG-N findings by assay platforms.^11^

The long duration of follow-up is a particular strength of this study. The study also used objective case ascertainment and validated antibody tests, and was sufficiently large to enable exploration of study outcomes in important population subgroups. It is a limitation of the study, however, that most cases were ascertained during the first phase of the study, and as such those with the longest follow-up in the second phase were identified mostly by antibody tests (PCR testing was not routinely performed during the early stages of the pandemic). More widespread PCR testing would have identified some cases who did not seroconvert, which may have reduced further the proportion with undetectable antibody levels during follow-up.^15^ Another limitation of the study was that severity of infection, which may affect the persistence of antibodies, was not assessed.^16^ Further, individuals with severe disease may have been less likely to return a sample, and as such the findings should be extrapolated with caution to those with severe infections, although this will have accounted for only a small proportion of cases.^17^ This study excluded individuals with evidence of reinfection; however, as the pandemic continues and reinfections increase, the effect on antibody levels in this context (including any difference in effect by major variants, such as Delta and Omicron) needs further study.^18^ It would also be valuable to understand further the effect of vaccination on immune response among those with and without natural infection, which was beyond the scope of the study.^19^

In conclusion, although antibody levels are detectable in the vast majority of individuals following infection over a 6-month period, there was an appreciable reduction over 18 months, with over a quarter of individuals having undetectable levels of IgG-N antibodies at this time. This waning of antibody levels over time supports the growing evidence on the limited long-term protection following natural infection, and the importance of vaccine schedules to increase antibody levels over the longer term. It also indicates that the use of IgG-N in population surveys is likely to substantially underestimate past infection at longer durations of follow-up.
