# Supplementary material for: Persistence of SARS-CoV-2 antibodies over 18 months following infection: UK Biobank COVID-19 Serology Study
Source: J Epidemiol Community Health. 2023 Nov 3;78(2):105–8. doi: 10.1136/jech-2023-220569 (PMC10850672; doi:10.1136/jech-2023-220569)
Supplement: Supplementary data [file jech-2023-220569supp001.pdf]

Supplementary Materials

Table S1: Characteristics of study participants

	All participants recruited		All participants who returned sample		Participants in IgG-S analyses		Participants in IgG-N analyses	
	**N**	**%**	**N**	**%**	**N**	**%**	**N**	**%**
**Age, years**								
<30	2 291	11.3	2049	11	228	15	126	7
30-39	3 169	15.7	2898	15	243	16	168	10
40-49	2 514	12.4	2340	12	181	12	143	8
50-59	3 928	19.5	3721	20	338	22	505	29
60-69	4 071	20.2	3881	21	279	18	460	26
70+	4 222	20.9	3998	21	245	16	347	20
**Gender**								
Men	8 877	44	8217	44	636	42	764	44
Women	11 318	56	10670	57	878	58	985	56
**Ethnicity**[a]								
White	17 626	87.5	16476	87	1 241	82	1 391	80
Other ethnicity	2 526	12.5	2372	13	271	18	354	20
**Townsend Deprivation Index**[b]								
Less Deprived	6 971	34.5	6576	35	453	30	571	33
Average	8 249	40.8	7728	41	590	39	715	41
More Deprived	4 975	24.6	4583	24	471	31	463	27
**UK Region**								
East Midlands	1 201	5.9	1133	6	76	5	90	5
East of England	938	4.6	889	5	53	4	60	3
London	6 059	30	5631	30	637	42	663	38
North East	832	4.1	780	4	44	3	63	4
North West	2 175	10.8	2035	11	167	11	205	12
Scotland	1 227	6.1	1168	6	60	4	70	4
South East	2 604	12.9	2441	13	166	11	193	11
South West	1 379	6.8	1301	7	65	4	87	5
Wales	791	3.9	742	4	37	2	56	3
West Midlands	1 407	7	1301	7	113	8	123	7
Yorkshire and The Humber	1 582	7.8	1466	8	96	6	139	8
**All**	20 195	100	18 887	100	1514	100	1 749	100

IgG-S antibody analyses restricted to SARS-CoV-2 cases with one or more IgG-S tests following diagnosis. IgG-N antibody analyses restricted to SARS-CoV-2 cases with IgG-N test following diagnosis. [a]Excludes participants of unknown ethnicity. [b]Area-level derived measure of socio-economic deprivation (categories are defined as: <-2 (less deprived), -2 to <2

Table S2: Proportion of SARS-CoV-2 cases seropositive for IgG-S antibodies at 6 months following infection

	SARS-CoV-2 cases, n	Positive for IgG-S antibodies, n	Proportion positive for IgG-S antibodies, % (95% CI)	χ2
**Age, years**				
<30	140	125	89.3% (82.9% - 93.9%)	
30-39	181	169	93.4% (88.7% - 96.5%)	
40-49	138	123	89.1% (82.7% - 93.8%)	
50-59	256	239	93.4% (89.6% - 96.1%)	
60-69	210	194	92.4% (87.9% - 95.6%)	
70+	175	157	89.7% (84.2% - 93.8%)	0.44
**Gender**				
Female	646	599	92.7% (90.4% - 94.6%)	
Male	454	408	89.9% (86.7% - 92.5%)	0.09
**Ethnicity[a]**				
White	890	812	91.2% (89.2% - 93.0%)	
Other ethnicity	208	193	92.8% (88.4% - 95.9%)	0.47
**Townsend Deprivation Index[b]**				
Less deprived	338	314	92.9% (89.6% - 95.4%)	
Average	422	386	91.5% (88.4% - 94.0%)	
More deprived	340	307	90.3% (86.6% - 93.2%)	0.47
**All**	1 100	1 007	91.5% (89.7% - 93.1%)	

Analyses among 1100 SARS-CoV-2 cases who had an IgG-S antibody test between 150-180 days following infection (mean 164 days; see Figure 2). [a]Excludes 2 participants of unknown ethnicity. [b]Area-level derived measure of socio-economic deprivation (categories are defined as: <-2 (less deprived), -2 to <2 (average), 2+ (more deprived).

Table S3: Proportion of SARS-CoV-2 cases seropositive for IgG-N antibodies at 18 months after infection

	**SARS-CoV-2 cases, n**	**Positive for IgG-N antibodies, n**	**Proportion positive for IgG-N antibodies, % (95% CI)**	**χ2**
**Age, years**				
<30	101	66	65.3% (55.2% - 74.5%)	
30-39	149	107	71.8% (63.9% - 78.9%)	
40-49	132	94	71.2% (62.7% - 78.8%)	
50-59	236	178	75.4% (69.4% - 80.8%)	
60-69	213	158	74.2% (67.8% - 79.9%)	
70+	191	139	72.8% (65.9% - 79.0%)	0.54
**Gender**				
Female	598	434	72.6% (68.8% - 76.1%)	
Male	433	311	71.8% (67.3% - 76.0%)	0.79
**Ethnicity[a]**				
White	833	591	70.9% (67.7% - 74.0%)	
Other ethnicity	196	152	77.6% (71.1% - 83.2%)	0.06
**Townsend Deprivation Index[b]**				
Less deprived	322	232	72.0% (66.8% - 76.9%)	
Average	415	295	71.1% (66.5% - 75.4%)	
More deprived	294	218	74.1% (68.7% - 79.1%)	0.66
**All**	1 031	745	72.3% (69.4% - 75.0%)	

Analyses among 1031 SARS-CoV-2 cases who had an IgG-N antibody test between 450-630 days following infection (i.e. the last time period analysed; see Figure 2) [a]Excludes 2 participants of unknown ethnicity. [b]Area-level derived measure of socio-economic deprivation (categories are defined as: <-2 (less deprived), -2 to <2 (average), 2+ (more deprived).

Figure S1: Antibody persistence to the N antigen over an 18 month period in days since the first positive PCR test

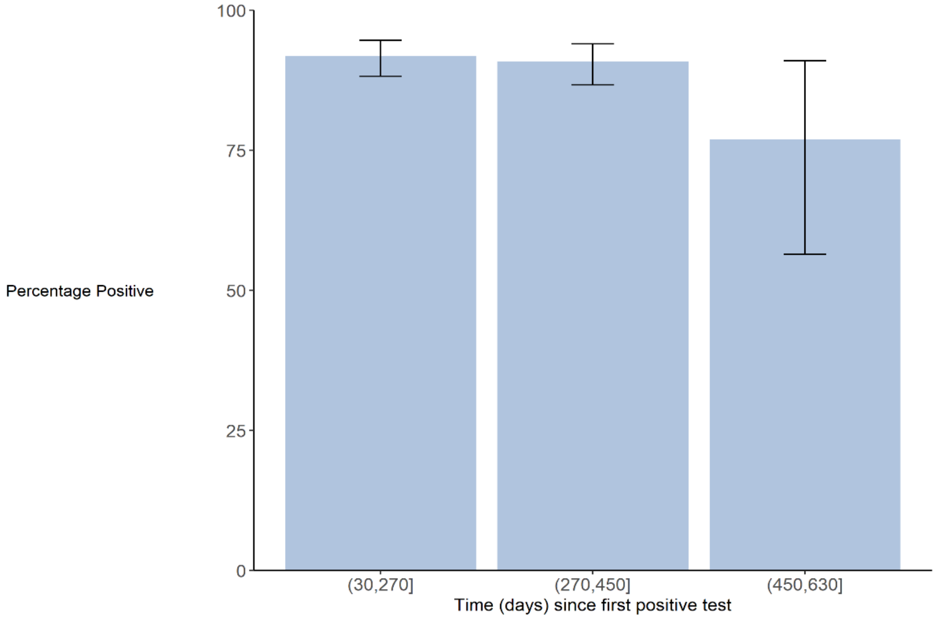

Total, n	306	262	26
Positive, n	281	238	20
%	91.8%	90.8%	76.9%
95% CI	88.2%-94.6%	86.7%-94.0%	56.4%-91.0%
Mean,days	92	362	540

Analyses among 674 SARS-CoV-2 cases with IgG-N test following diagnosis (78 individuals are omitted from the plot as they have a duration of follow up less than 30 days and a further 2 individuals are omitted from the plot as they exceed 630 days follow up post infection). Proportions are % (95% CI) of IgG-N seropositive cases among participants that returned a valid test during each period; participants provided one IgG-N test only. Figure shows the observed proportion with 95% confidence interval. The table below the figure shows the

number of participants in each bar, as well as the number positive and the % positive with 95% confidence interval. Date of infection was determined by PCR test. (30-270) represents upto 9 months, (270-450) represents 9 to 15 months, and (450-630] represents 15 to 21 months.
